# Proteasome inhibitor lactacystin enhances cisplatin cytotoxicity by increasing endoplasmic reticulum stress-associated apoptosis in HeLa cells

**DOI:** 10.3892/mmr.2014.2683

**Published:** 2014-10-16

**Authors:** YE XU, DI LI, LINCHUAN ZENG, CHUNYAN WANG, LILI ZHANG, YAN WANG, YANG YU, SHIBING LIU, ZHIXIN LI

**Affiliations:** 1 Medical Research Laboratory, Jilin Medical College, Changchun, Jilin 130021, P.R. China; 2 Department of Histology and Embryology, Jilin Medical College, Changchun, Jilin 130021, P.R. China; 3 Department of Urology, Xijing Hospital, Fourth Military Medical University, Xi’an, Shaanxi 710032, P.R. China

**Keywords:** lactacystin, cisplatin, apoptosis, ER stress, cervical cancer

## Abstract

Cisplatin is commonly used as a therapeutic agent, despite its known adverse side effects and the occurrence of drug resistance. The development of novel methods for combination therapy with cisplatin is required in order to circumvent these limitations of cisplatin alone. The proteasome inhibitor lactacystin (LAC) has been indicated to produce anti-tumor effects, and has previously been used as an antitumor agent in cancer treatment research; however, its effects in combination with cisplatin treatment are unknown. In the current study, the effects of LAC in combination with cisplatin treatment were investigated in HeLa human cervical cancer (HCC) cells. The results demonstrated that cisplatin treatment inhibited cell growth and induced cell apoptosis. HeLa cell exposure to cisplatin induced endoplasmic reticulum (ER) stress-associated apoptosis, and LAC treatment increased levels of cell apoptosis and the activation of caspase-3. Specifically, LAC treatment increased the cisplatin-induced expression of PDI, GRP78, CHOP, cleaved caspase-4 and cleaved caspase-3. Together, these data indicate that LAC is able to enhance cisplatin cytotoxicity by increasing ER stress-associated apoptosis in HeLa cells.

## Introduction

Cisplatin (cis-diamminedichloroplatinum II; CDDP) is one of the most effective chemotherapeutic agents and is widely used for the treatment of solid tumors. The side effects and acquired drug resistance that occur during cisplatin treatment limit its clinical use (1–3). The primary cellular target of cisplatin is considered to be nuclear DNA. Cisplatin-induced DNA damage activates various signaling pathways to promote cell death predominantly by inducing apoptosis (4–6). A number of studies have identified that cisplatin can induce endoplasmic reticulum (ER) stress and nucleus-independent apoptotic signaling (7–10).

Various physiological and pathological conditions may lead to ER stress, which results in an accumulation of unfolded or misfolded proteins in the ER lumen (11,12). This cellular stress subsequently causes an activation of the unfolded protein response (UPR), which induces the expression of chaperones and proteins involved in the recovery process. Severe ER stress can lead to cell death, commonly by the activation of intrinsic apoptosis (13,14). The accumulated unfolded or misfolded proteins in the ER are marked for degradation by the ubiquitin-proteasome or autophagy-lysosome pathway (15,16). Previous studies have demonstrated that inhibitors of autophagy, such as 3-methyladenine and chloroquine, effectively enhance the cytotoxicity of chemotherapeutic agents such as cisplatin and S1 by increasing ER stress (17–20). Thus, in the present study, the effect of the proteasome inhibitor lactacystin (LAC) on cisplatin cytotoxicity was assessed. LAC covalently binds to the N-terminal threonine of the 20S proteasome subunit X, and irreversibly modifies all catalytic β subunits. LAC inhibits proteases such as cathepsin A and tripeptidyl peptidase II (21–24).

In the present study, it was hypothesized that the use of LAC would increase ER stress-associated apoptosis induced by cisplatin in HeLa human cervical cancer (HCC) cells. HeLa cells were treated with cisplatin, LAC or a combinational therapy incorporating the two drugs simultaneously, and the subsequent effects were analyzed by MTT assay, protein and RNA expression analyses.

## Materials and methods

### Cell culture

HeLa human cervical cancer cells were cultured at 37°C in an atmosphere of 5% CO_2_ and 95% air, in Iscove’s modified Dulbecco’s medium (IMDM; Life Technologies, Grand Island, NY, USA) supplemented with 10% fetal bovine serum (FBS; Gibco Life Technologies, Carlsbad, CA, USA)and 100 U/ml penicillin and 100 μg/ml streptomycin, prior to use in all experiments. The cells were divided into four groups as follows: Non-treated cells; cisplatin-treated cells (5 μg/ml); LAC-treated cells (10 μM); Cisplatin (5 μg/ml) and LAC (10 μM)-treated cells. Cisplatin and LAC were purchased from Sigma-Aldrich (St. Louis, MO, USA)

### MTT assay

Cell viability was determined by MTT assay. HeLa cells, during the exponential growth phase, were seeded into 96-well culture plates in 100 μl IMDM at a density of 1×10^4^ cells/well. After 24-h incubation, the indicated dose of cisplatin (5 μg/ml) and/or LAC (10 μM) was added for 12-h incubation in four parallel wells. MTT assays (Beyotime Institute of Biotechnology, Haimen, China) were performed as follows: 20 μl MTT solution (5 mg/ml in PBS) was added to the cells for 4 h, after which, 150 μl dimethyl sulfoxide (Beijing Chemical Industry Co., Ltd., Beijing, China) was added to each well. The cells were agitated for 10 min, prior to absorbance measurements at 570 nm using a Microplate Reader (Bio-Rad Laboratories, Hercules, CA, USA). The growth inhibition rate was calculated as % inhibition = 1 − absorbance of experimental group/absorbance of control group × 100. The mean value of the four replica wells was calculated for each treatment group.

### Western blotting

Whole-cell protein extracts from HeLa cells were prepared with cell lysis buffer (50 mM Tris-HCl, pH 7.5; 150 mM NaCl; 1 mM Na2EDTA; 1 mM EDTA; 1% Triton; 2.5 mM sodium pyrophosphate; 1 mM β-glycerophosphate; 1 mM Na_3_VO_4_; 1 mM NaF; 1 μg/ml leupeptin; and 1 mM PMSF) for western blotting. The protein extracts were quantified using a Bio-Rad Protein Assay kit (Bio-Rad Laboratories). For Western blot analysis, protein lysates (30–50 μg) were separated by 12% SDS-PAGE and transferred onto Immobilon-P Membranes (EMD Millipore, Billerica, MA, USA). The membranes were blocked with 5% non-fat dry milk in buffer (10 mM Tris-HCl, pH 7.6; 100 mM NaCl; and 0.1% Tween 20) for 2 h at room temperature and then incubated with the appropriate primary antibodies, including the monoclonal rabbit anti-human Ub and monoclonal rabbit anti-human caspase-3 (1:1,000 dilutions; Epitomics, Burlingame, CA, USA), monoclonal mouse anti-human PDI, monoclonal mouse anti-human p62, monoclonal mouse anti-human Grp78, monoclonal mouse anti-human CHOP, polyclonal rabbit anti-human caspase-4, monoclonal rabbit anti-human caspase-3 and monoclonal mouse anti-human β-actin (1:1,000 dilutions; Santa Cruz Biotechnology, Inc., Santa Cruz, CA, USA) overnight at 4°C, followed by incubation with horseradish peroxidase-conjugated secondary antibody (Hangzhou HuaAn Biotechnology Co.. Ltd., HangZhou, China) at 1:2,000 dilution for 1.5 h at room temperature. The immunoreactive bands were visualized by the diaminobenzidine (Sigma-Aldrich) coloration method. The representative bands were measured using a Tanon Gel Imaging System (Tanon Science and Technology Co., Ltd., Shanghai, China) and analyzed. The protein expression levels were normalized to actin and the ratios of the normalized protein are presented as the means ± standard deviation from three independent experiments. The protein levels were quantified by densitometry using Quantity One 1-D Analysis Software (Bio-Rad Laboratories).

### Immunofluorescence staining and confocal laser microscopy

HeLa cells were cultured on coverslips overnight, and were then treated with cisplatin (5 μg/ml) and/or LAC (10 μM) for 12 h. The cells were then fixed with 4% paraformaldehyde (Beijing Chemical Industry Co., Ltd.), stained with the Hoechst 33342 nuclear stain (2 μg/ml; Sigma-Aldrich) for 30 min, washed with phosphate-buffered saline (PBS; Beijing Zhongshan Golden Bridge Biological Technology Co., Ltd., Beijing, China), and examined using an Olympus FV1000 confocal laser microscope (Olympus Corporation, Tokyo, Japan) to reveal chromatin condensation. The expression levels of active caspase-3 and γ-H_2_AX were examined by indirect immunofluorescence methods. Briefly, cells were cultured on coverslips overnight and treated with cisplatin (5 μg/ml) and/or LAC (10 μM) for 12 h. The cells were then rinsed 3 times with PBS prior to fixation with 4% paraformaldehyde for 20 min. The cells were then permeabilized with 0.1% Triton X-100 (Beijing Dingguo Changsheng Biotechnology Co., Ltd., Beijing, China) for 5 min and blocked with bovine serum albumen (Beijing Dingguo Changsheng Biotechnology Co., Ltd.), prior to incubation with primary antibodies against active caspase-3 (Epitomics) and γ-H_2_AX (Cell Signaling Technology, Inc., Danvers, MA, USA) (1:100 dilution) overnight at 4°C. The cells were then incubated with Alexa Fluor543/488-conjugated secondary antibody (1:400; Invitrogen Life Technologies, Carlsbad, CA, USA) for 1 h, then stained with the Hoechst 33342 (2 μg/ml) for 2 min, and washed with PBS 3 times. The cells were mounted and examined by confocal laser microscopy.

### Statistical analysis

Data are representative of three independent experiments each conducted in triplicate. Statistical analysis of the data was performed using one-way analysis of variance. Tukey’s post-hoc test was used to determine the significance for all pairwise comparisons of interest. P<0.05 was considered to indicate a statistically significant difference.

## Results

### LAC potentiates cell growth inhibition induced by cisplatin

**Figure 1:**
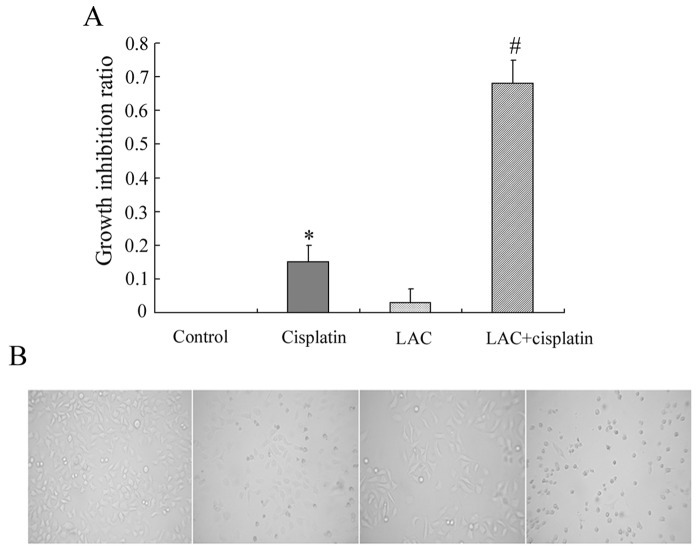
LAC increases the growth inhibition rate induced by cisplatin in HeLa human cervical cancer cells. HeLa cells were treated with cisplatin (5 μg/ml) and/or LAC (10 μM) for 12 h. (A) Cell viability was determined by MTT assay. (B) Images were captured with an inverted phase contrast microscope at a magnification of ×100. Data are presented as the mean ± standard deviation, n=3. ^*^P<0.05 vs*.* control, ^#^P<0.05 vs*.* cisplatin. LAC, lactacystin.

Based on previous studies, HeLa cells were treated with the indicated doses of cisplatin and/or LAC for 12 h, and then the growth inhibition was examined by MTT assay. It was observed that cisplatin inhibited the growth of HeLa cells. MTT assay indicated that LAC alone exerted no significant effect on cell viability, and LAC treatment enhanced the cytotoxic effect of cisplatin when administered in combination (Fig. 1A). Changes to cellular morphology were observed under a inverted phase contrast microscope. Compared with the controls, round and fragile cells were detected in the cisplatin treatment group. The number of round and fragile cells was increased in the group treated with cisplatin combined with LAC (Fig. 1B).

### LAC increases cisplatin-induced cell apoptosis

**Figure 2:**
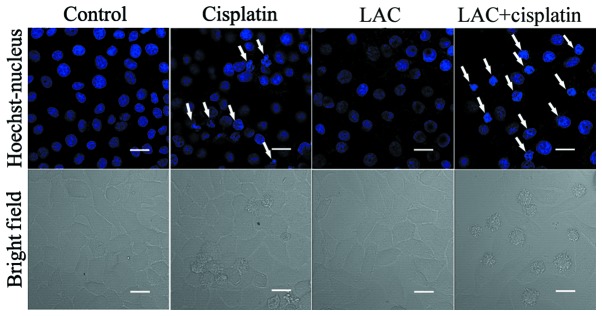
LAC increases cisplatin-induced apoptosis in HeLa human cervical cancer cells. HeLa cells were treated with cisplatin (5 μg/ml) and/or LAC (10 μM) for 12 h then stained with Hoechst 33342. Cell morphology was observed by confocal microscopy (Scale bar, 20 μm, arrows, apoptotic cells; magnification, ×800). LAC, lactacystin.

The levels of apoptosis were analyzed in order to whether LAC may potentiate the apoptosis induced by cisplatin in HeLa cells. Apoptotic chromatin condensation was analyzed with Hoechst 33342 staining and confocal microscopy. As compared with the control cells, cisplatin-induced apoptotic chromatin condensation was clearly observed. As compared with the cisplatin-treated group, the cells treated with both cisplatin and LAC exhibited a marked increase in the levels of apoptotic chromatin condensation (Fig. 2).

**Figure 3:**
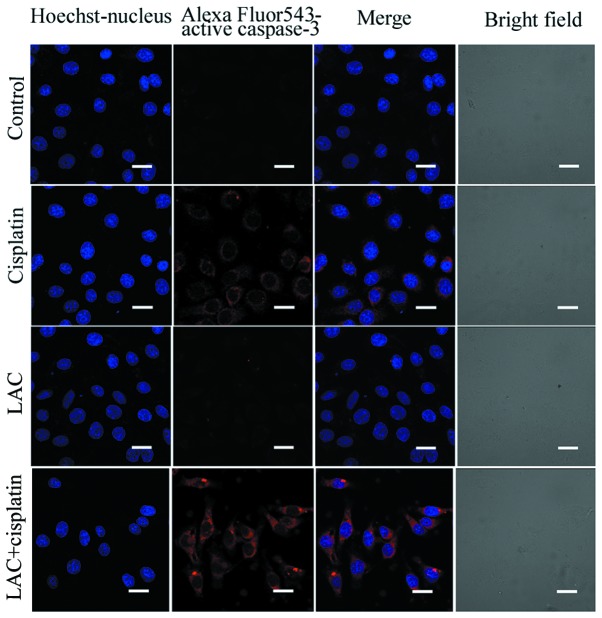
LAC increases the expression of active caspase-3 in HeLa human cervical cancer cells. HeLa cells were treated with cisplatin (5 μg/ml) and/or LAC (10 μM) for 12 h. The expression of active caspase-3 was detected by confocal microscopy with varying treatments (scale bar, 20 μm; magnification, ×800). LAC, lactacystin.

Caspase-3 functions as an executioner molecule during apoptosis, and its activation reflects the initiation of apoptosis. Using confocal microscopy, the activation of caspase-3 was detected in the cisplatin-treated cells and those treated with cisplatin combined with LAC (Fig. 3). The expression of caspase-3, indicated by indirect red fluorescence, was stronger in the cells treated with cisplatin combined with LAC compared with cells treated with cisplatin alone, indicating that the combined treatment increased caspase-3 activation. These results indicate that LAC can efficiently increase the apoptosis induced by cisplatin in HeLa cells.

### LAC increases cisplatin-induced ER stress

**Figure 4:**
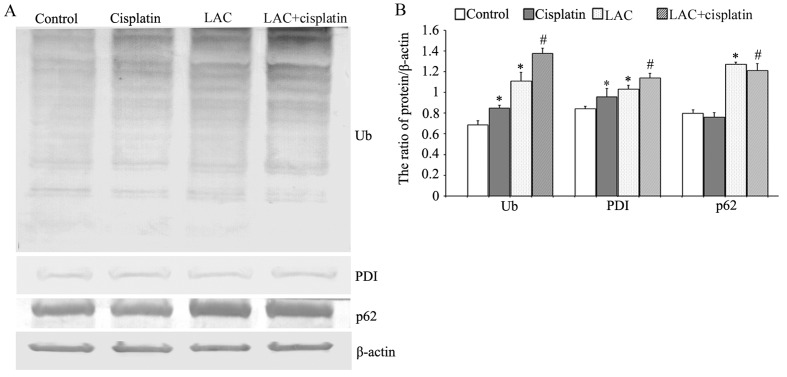
LAC increased cisplatin-induced ER stress in HeLa human cervical cancer cells. (A) HeLa cells were treated with cisplatin (5 μg/ml) and/or LAC (10 μM) for 12 h. Western blot analysis of Ub, PDI and p62. (B) Quantification of the protein levels. Data are presented as the mean ± standard deviation, n=3. ^*^P<0.05 vs. control, ^#^P<0.05 vs. cisplatin. LAC, lactacystin; ER, endoplasmic reticulum; Ub, ubiquitinated protein; PDI, protein disulfide isomerase.

Previous studies have indicated that cisplatin can induce ER stress by misfolded protein accumulation. Misfolded proteins may be ubiquitinated, marking them for degradation (17). Therefore the expression levels of ubiquitin (Ub), ER chaperone protein disulfide isomerase (PDI; which reflects the occurrence of ER stress), and p62 (an adaptor that transports the ubiquitinated proteins) were analyzed by western blotting. It was observed that cisplatin and LAC treatments induced a higher level of Ub. The combination of cisplatin and LAC markedly increased the ubiquitination of proteins. Cisplatin and LAC increased the expression of PDI compared with the control cells, and combination treatment increased the expression of PDI compared with the cisplatin group. In addition, the protein level of p62 showed a slight reduction following cisplatin treatment, whilst LAC treatment increased its level in cells treated with LAC alone or when combined with cisplatin (Fig. 4). These results indicate that LAC can increase cisplatin-induced ER stress in HeLa cells.

### LAC increases cisplatin-induced ER stress-associated apoptosis

**Figure 5:**
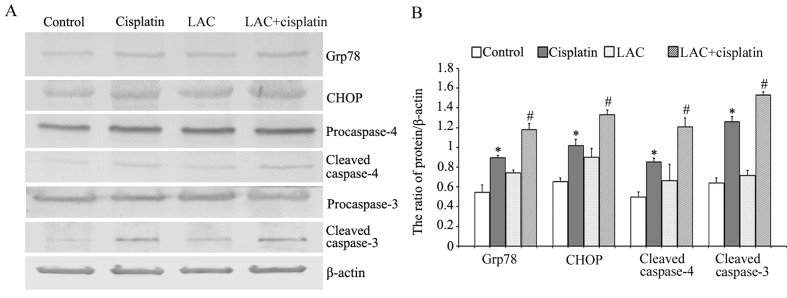
LAC increased cisplatin-induced endoplasmic reticulum stress-associated apoptosis in HeLa human cervical cancer cells. (A) HeLa cells were treated with cisplatin (5 μg/ml) and/or LAC (10 μM) for 12 h. Western blot analysis of grp78, CHOP, caspase-4, cleaved caspase-4, caspase-3 and cleaved caspase-3. (B) Quantitation of the protein levels. Data are presented as the mean ± standard deviation, n=3. ^*^P<0.05 vs. control, ^#^P<0.05 vs. cisplatin. LAC, lactacystin; grp78, glucose-regulated protein-78; CHOP, 153/C/EBP homology protein.

Glucose-regulated protein-78 (Grp78) is an ER chaperone protein, which can be upregulated by ER stress (18). The growth-arrest and DNA-damage-inducible gene, 153/C/EBP homology protein (GADD153/CHOP), is involved in ER stress-induced cell death; sustained ER stress induces caspase-mediated apoptosis (17,18). Caspase-4 is an ER-resident caspase that, similar to murin caspase-12, is processed in response to ER stress and is activated during ER stress-induced apoptosis. Using western blot analysis, it was observed that cisplatin induced the upregulation of Grp78, CHOP, and cleaved caspases 3 and 4. Compared with cisplatin, LAC increased the expression levels of all these proteins (Fig. 5). These results indicate that LAC increased cisplatin-induced ER stress-associated apoptosis.

### LAC does not enhance the level of cisplatin-induced DNA double-strand breaks (DSB)

**Figure 6:**
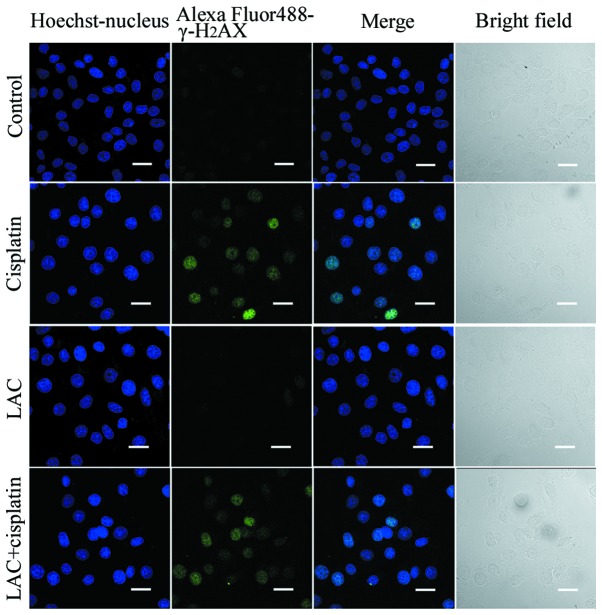
NH_4_Cl does not increase the phosphorylation of H_2_AX. HeLa cells were treated with cisplatin (5 μg/ml) and/or LAC (10 μM) for 12 h. The expression of γ-H_2_AX was detected by confocal microscopy with varying treatments (Scale bar, 20 μm; magnification, ×800). LAC, lactacystin.

Cisplatin acts to damage DNA and inhibit DNA synthesis, thus resulting in cancer-cell death (6). Therefore it was hypothesized in the current study that LAC may increase DNA breakage induced by cisplatin. DNA DSB are able to induce phosphorylation of H_2_AX at a conserved serine 139 residue in the C terminal region, leading to the formation of γ-H_2_AX. This molecule is commonly used as a DNA DSB marker. Using confocal microscopy, the expression of γ-H_2_AX, reported by indirect green fluorescence, was visualized in both the cells treated for 12 h with cisplatin and cisplatin combined with LAC (Fig. 6). These results indicate that LAC does not enhance the DSB induced by cisplatin in HeLa cells.

## Discussion

Cisplatin has been used as a chemotherapeutic agent in the clinical setting for many years, with positive effects against numerous types of solid tumors, including cervical cancer (1). The produced side-effects and drug resistance of cisplatin limit its use. The predominant pathway of cell death that is induced by cisplatin is a caspase-dependent intrinsic apoptotic pathway involving the mitochondria and ER (6,10,25).

The ER has been previously reported to be a target of cisplatin-induced apoptosis (17,18). It was demonstrated that cisplatin was able to induce ubiquitinated protein accumulation and lead to ER stress in HeLa and SKOV3 cells (17,18). The following three ER sensors have been identified: PKR-like ER kinase, inositol requiring enzyme 1 and activating transcription factor 6 in UPR induction (26). Upon induction of ER stress is induced, the cell upregulates several ER resident chaperones, such as GRP78 and PDI, to re-establish ER homeostasis. Simultaneously, the misfolded or unfolded proteins are transported to be degraded by the ubiquitin-proteasome or autophagy-lysosome pathways (11,12,26). P62 is a multifunctional molecule, functioning as an adaptor to bind ubiquitinated proteins and transport them for degradation. Once the proteins are degraded, bound p62 molecules are also degraded (27,28). Sustained and unabated ER stress induces the activation of apoptosis. CHOP (GADD153) is a member of the C/EBP family of bZIP transcription factors, and its expression is upregulated by ER stress. CHOP participates in ER-stress-corrective actions through induction or suppression of a number of genes. Prolonged activation of CHOP can trigger apoptosis in cells under certain conditions (29,30).

In the present study, it was demonstrated that cisplatin treatment inhibited cell growth and induced cell apoptosis in HeLa cells. In addition, exposure to cisplatin increased the expression of Ub, PDI and GRP78 and upregulated the level of CHOP and cisplatin treatment induced the activation of caspase-4 and caspase-3. Together, these findings indicate that cisplatin can induce ER stress-associated apoptosis in human cervical cancer HeLa cells. LAC treatment combined with cisplatin potentiated the effects of cisplatin alone. DNA damage is considered an indicator of apoptosis in cisplatin cytotoxicity. H_2_AX phosphorylation occurs in response to cisplatin-induced DNA lesions. The formation of γ-H_2_AX is a useful indicator of cisplatin-induced DNA damage (31). Thus, the changes to γ-H_2_AX formation in cells treated with cisplatin combined with LAC was investigated in the present study. However, there was no difference between cells treated with cisplatin alone and those treated with cisplatin combined with LAC. These results indicate that LAC enhanced cisplatin cytotoxicity by increasing ER stress-associated apoptosis, rather than by increasing DNA damage.

In conclusion, it was demonstrated that cisplatin treatment induced ER stress-associated apoptosis in human cervical cancer HeLa cells. LAC treatment combined with cisplatin increased the cell growth inhibition rate, cell apoptosis and activation of caspase-3. Additionally, LAC treatment increased the cisplatin-induced expression levels of PDI, GRP78, CHOP, cleaved caspase-4 and cleaved caspase-3. These data indicate that LAC is able to enhance cisplatin cytotoxicity by increasing ER stress-associated apoptosis, and it has the potential to improve the results of cisplatin chemotherapy.
